# Species characteristics and cultural value of stone wall trees in the urban area of Macao

**DOI:** 10.1038/s41598-022-05522-2

**Published:** 2022-01-28

**Authors:** Meisi Chen, Songyi Huang, Zheng Chen, Yemiao Xing, Fuwu Xing, KunFong Leong, Yaonan Yang, Yuan Qiu, Xinsheng Qin

**Affiliations:** 1 College of Forestry and Landscape Architecture, South China Agricultural University, Guangzhou, China; 2 Guangzhou Landscaping Company, Guangzhou, China; 3 School of Architecture, South China University of Technology, Guangzhou, China; 4 South China Botanical Garden, The Chinese Academy of Sciences, Guangzhou, China; 5 Association for the Promotion of Landscaping and Greening in Macao, Guangzhou, China; 6 Brilliant Landscaping & Gardening Co., Ltd., Guangzhou, China

**Keywords:** Biodiversity, Plant development

## Abstract

The stone walls remaining in the highly urbanized Macao area provide a special habitat for plants, repair the fragmentation of the habitat caused by urbanization, and enhance the urban biodiversity. The main object of this survey are stone wall trees in Macao. The species composition, frequency of occurrence and distribution were ascertained, and the feasibility of establishing stone wall tree landscape trail systems was discussed. The results showed that there were 96 stone wall trees in Macao. Among them, 47.9% of the total in the historical center of Macao. In addition, research and analysis on the species composition, life form, frequency and similarity of the associated plants of stone wall trees were analyzed. The survey found that there were 101 species of companion plants, and herbaceous plants had the greatest growth advantage. Most of the companion species were selective and incidental to the stone wall habitat; the similarity of the companion plants in different habitats was less than 0.25, showing that the stone wall was conducive to species diversity. The results of this research aim to explore planning strategies for holistic conservation of stone wall landscape, and provide a theoretical basis for studying the biodiversity of special habitats in Macao.

## Introduction

Cities characterized by high-density, such as Hong Kong and Macao, have caused severe city problems, like crowded urban space, insufficient ecological green space, and deterioration in quality of residents' life^1^. These urged people to pay more attention to the development of vertical greening space. The greening form of three-dimensional space can hopefully reconcile the contradictions between city development and ecological environment. Meanwhile, walls left or under construction in the city can provide growth potential for some biological groups.

The stone wall in the city refers to the vertical or near vertical surface including retaining wall, enclosure wall, ancient city wall, etc., built by stones, bricks, mortars, concrete and other materials^2^. Urban stone wall is one of the remaining natural habitats of the city. The organisms growing on walls can adapt to urban environment and human disturbance, thereby increasing the city biodiversity, functioning in adjusting local microclimate, and reflecting certain regional characteristics of the city^3^.

As early as the nineteenth century, some European scholars had begun to investigate the plants on historical sites like ancient buildings, ancient city walls, and so on. Through field research, some researches about the plants on walls or ancient buildings had been published successively in England, Italy, Rome, Poland, and Czech Republic^4^. In recent years, researches abroad on wall plants have focused on the relationship between wall habitat characteristics and plant diversity^5^. However, there is no clear definition of plants growing on artificial stones or gaps.

Wall plants were defined by Jim^6^ according to the stone walls he studies in Hong Kong. He thought that wall plants referred to plants with most roots penetrating into the wall surface, gap or within the wall boundary, excluding plants with most roots not within the wall boundary and plants not attached to the wall. Stone wall trees are consistent with the definition of wall plants put forward by Jim, which accounts for the similar origins of stone wall trees in Hong Kong and Macao.

In Chinese mainland, the earliest researches on wall plants mainly focused on the ancient city walls in Jingzhou City and Nanjing City. Lei et al.^7^ compiled the list of angiosperms on Jingzhou Ancient City Wall; Zhou et al.^8^ further studied the plant communities’ differences due to the slope direction in the north and south section of Jingzhou Ancient City Wall. Wang^9^ explored the propagation mechanism of vascular plants on the Ming Dynasty wall of Nanjing. In recent years, some researches on wall biodiversity and city habitat in Hong Kong, Macao, Pearl River Delta, Zhejiang, Chongqing, have been in an increasing trend. In terms of wall habitat, Wu ^10^proposed suggestions for plant configuration on the wall by comparing the plant diversity and spatial structure of urban plant walls with the ones of rural plant walls; Li et al.^11^ analyzed the reproduction characteristics of the stone wall plants, and compared the differences in species composition between cities in adjacent climate zones. The study of wall habitat began to focus on the connection between wall plants and surrounding ecological environment. Researches on wall plants in Hong Kong, Macao and the Pearl River Delta mostly focused on plant diversity. Zhang et al.^12^ studied the flora and life form of wall plants in Macao. The results showed that wall plants were most herbaceous species, and had the most distribution types of tropical Asia^13^. Xie et al.^14^ investigated and analyzed the drought tolerance, ornamental, and application of wall plants in the Pearl River Delta. The researches focused on the existing wall plants species and classified analysis.

As stone wall trees less distribute in the world and the traditional stone masonry technology has been lost, stone wall trees are becoming rarer. There are few studies on stone wall trees in Macao. This study is expected to acquaint the special components of urban forestry in Macao, and to provide some inspirations for the conservation of stone wall trees.

## Materials and methods

### Study area

**Figure 1:**
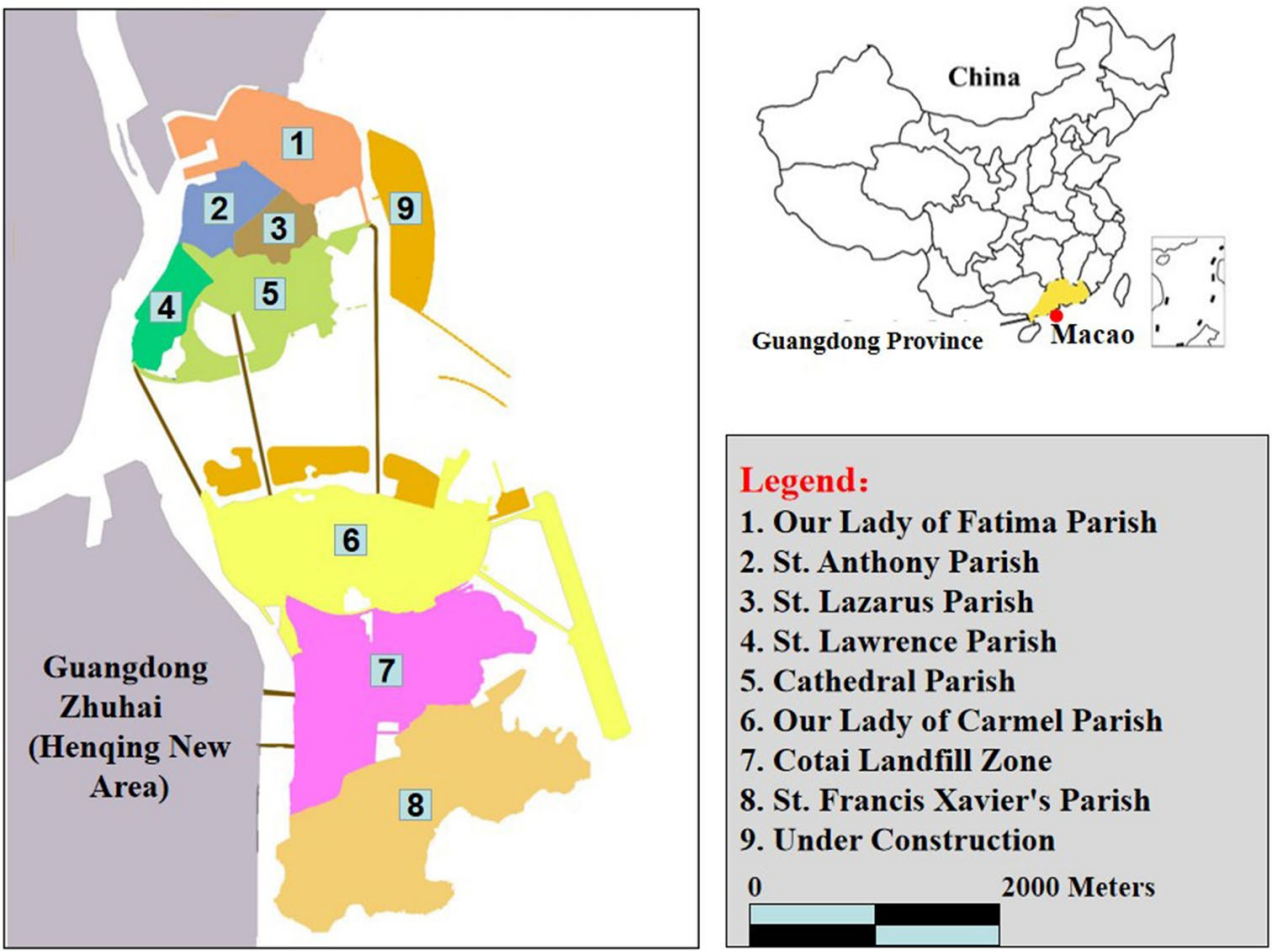
Location of the study area. (URL of the Chinese map: https://www.d-maps.com/m/asia/china/chine/chine48.gif; URL of the Macao map: https://www.d-maps.com/m/asia/china/macau/macau02.gif).

This study was conducted in Macao (22°06′39″ ~ 22°13′06″N, 113°31′45″ ~ 113°35′43″E), which locates on the coast of the South Sea, connects with Zhuhai city in the north, and connects Hong Kong in the east (Fig. 1). It is sub-tropical maritime climate with mean annual temperature of 14.6 °C and mean annual precipitation of more than 2000 mm^15^. There were 1508 species under 866 genera and 2016 families, including wild plants of 812 species under 525 genera and 158 families^16^.

### Analytical methods

We inspected the distribution of stone wall trees in Macao, and there were a total of 31 samples. According to the *Flora of China* and *The Flora of Macao*, the species was classified and identified. The main species identifiers were Professors Xing Fuwu and Qin Xinsheng, both of whom have conducted long-term field surveys in Hong Kong, Macao and Guangdong and are familiar with the species in the region. The study used excel and GIS for data analysis and statistical integration.

1. Proportion of occurrence frequency of plants = Number of occurrence of a certain specie/total number of stone wall trees × 100%
2. Jaccard Similarity Coefficient

A simpler mathematical expression of community similarity is the community coefficient of Jaccard^17^.

The calculation formula is:

$${\text{Sj}} = {\text{c}}/\left( {{\text{a}} + {\text{b}} + {\text{c}}} \right) \times {1}00\%$$

a: number of unique species recorded in the first sample; b: number of unique species recorded in the second sample. c:Species shared by both sites.

## Results

### Species composition of stone wall trees

#### Families and genera of stone wall trees

There were 96 stone wall trees belonging to 6 genera and 5 families in Macao. Among them, Moraceae and *Ficus* appeared the most frequently, both reaching 85 times, accounting for 88.5% (Table 1). It showed that Moraceae, a kind of tropical distribution family, was dominant in the stone wall trees communities, which meant that stone wall trees species in Macao appeared distinctly tropical nature^18^.

**Table 1:** Frequency of occurrence of stone wall Trees in different families and genera.

Family	Frequency of occurrence (%)	Genera	Frequency of occurrence (%)
Moraceae	88.5	*Ficus*	88.5
Ulmaceae	5.2	*Celtis*	4.2
Euphorbiaceae	3.1	*Bridelia*	3.1
Mimosaceae	2.1	*Leucaena*	2.1
Rosaceae	1.0	*Eriobotrya*	1.0
		*Trema*	1.0

#### Species of stone wall trees

**Figure 2:**
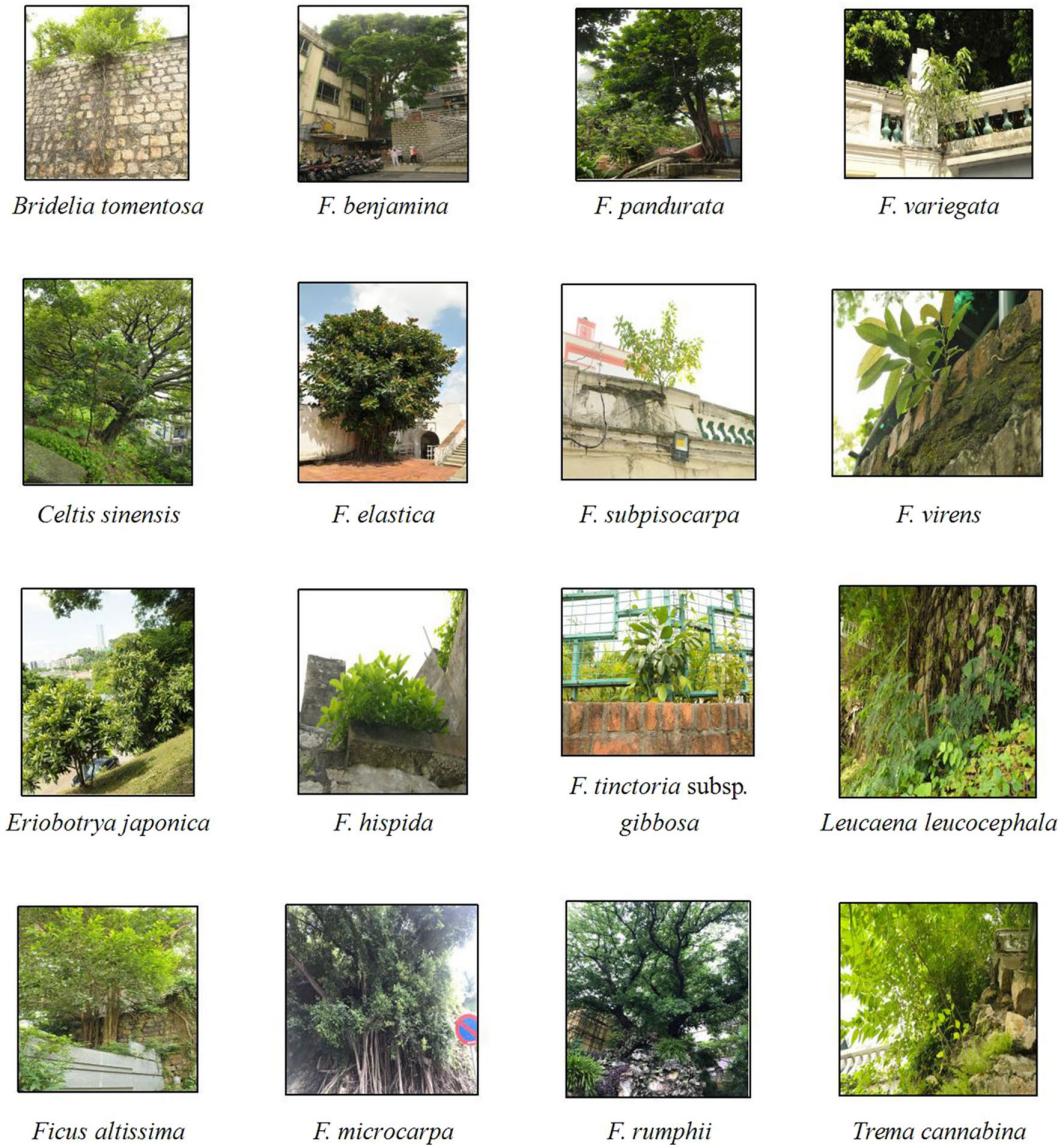
16 species of stone wall trees in Macao (photo was taken by Professor Qin Xingsheng).

There were 16 species of the stone wall trees in Macao including *Bridelia tomentosa*, *Celtis sinensis*, *Eriobotrya japonica*, *Ficus altissima*, *F. benjamina*, *F. elastica*, *F. hispida*, *F. microcarpa*, *F. pandurata*, *F. subpisocarpa*, *F. tinctoria* subsp*. gibbosa*, *F. rumphii*, *F. variegata*, *F. virens*, *Leucaena leucocephala*, and *Trema cannabina* (Fig. 2).

**Figure 3:**
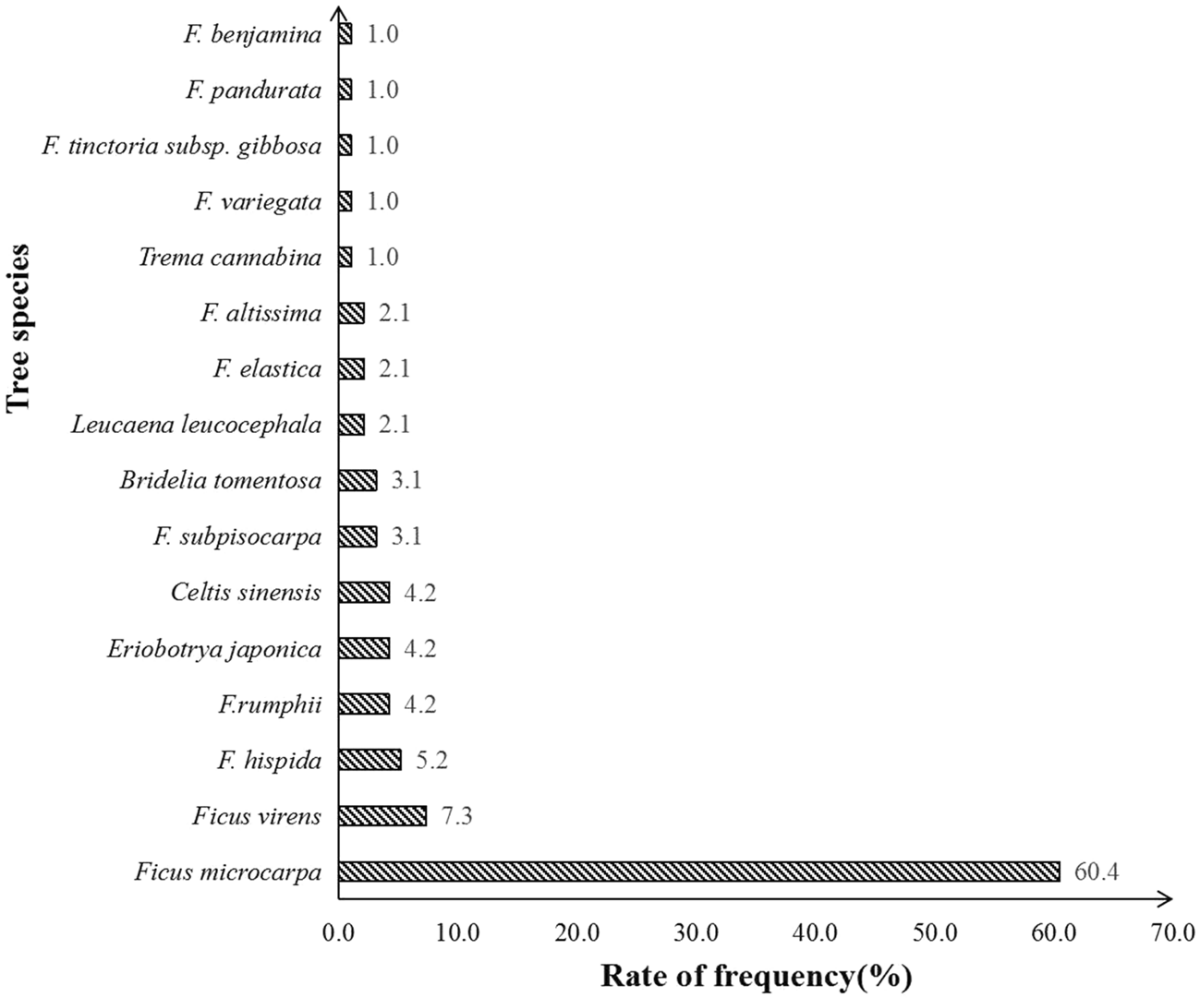
Frequency distribution of stone wall tree species in Macao.

Based on the frequency of occurrence of various tree species, the frequency was concentrated in the range of 1–5%. Among them, *Ficus microcarpa* had the highest frequency, reaching 58 times, with a frequency of 60.4% (Fig. 3). This tree species is robust, adaptable and fast growing, which is the main population of *Ficus*^19^.

### Stone wall trees in the historic center of Macao

The historic center of Macao, covering an area of about 2.8 km^2^, is the heartland of Macao's historical and cultural heritage, which plays a significant role in the cultural heritage around the world^18^. The historic center of Macao provides valuable historical and cultural resources that enable Macao to transform into a world tourism center^20^.

**Figure 4:**
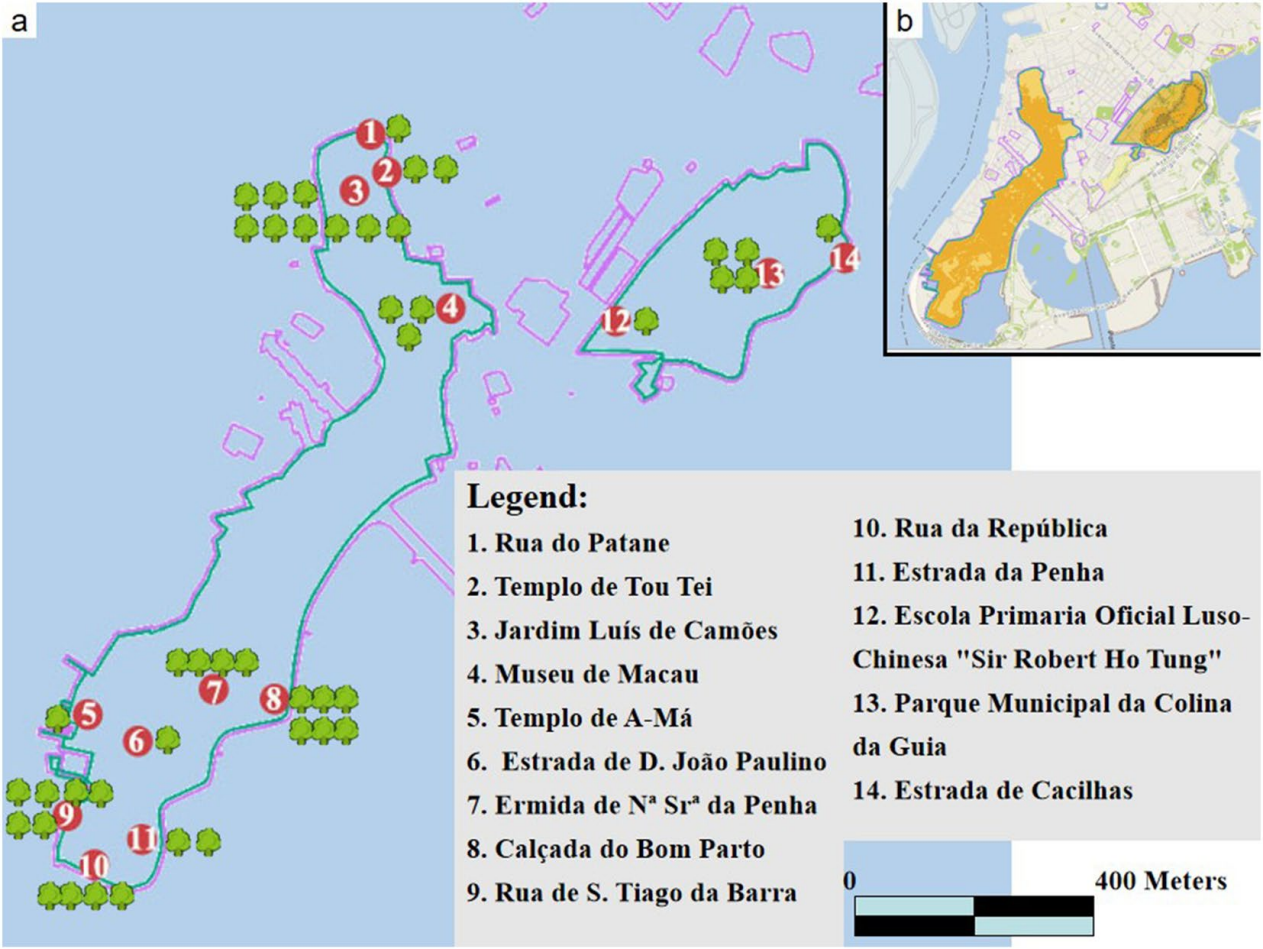
(**a**) Schematic diagram of distribution and number of stone wall trees in the historic Center of Macao. (**b**) Schematic diagram of historic center of Macao. (URL of the Macao map: https://www.d-maps.com/m/asia/china/macau/macau02.gif).

A total of 14 plots were located in the historic Center of Macao (Fig. 4), with 45 stone wall trees, accounting for 47.9% of the total number of trees in the survey. Among them, Jardim Luís de Camões has the largest number of 9 stone wall trees. The park, built in the mid-eighteenth century, is one of the oldest gardens in Macao and has the largest number of old trees in Macao. The park had provided good time and environmental conditions for the growth of stone wall trees.

According to Decree No. 56/84/M of the Macao Special Administrative Region Government Printing Department, immovable property that represents the creation of man, or the development of nature or technology and has cultural significance is considered tangible cultural property. The occurrence of the stone wall tree was inextricably linked to ancient wall-building techniques of that time, which was of great significance for the study of the technological development and ecological landscape of the historic center of Macao. The concept of "historic urban landscape" was proposed by Zhang Song^20^, who argued that cities were organisms in continuous evolution, emphasizing respect for the interrelationship between natural and man-made environments. The stone wall trees in the historic center of Macao have been associated with the local culture and ecology tightly and should be preserved as important urban landscape.

### Symbiotic relationship between tree and stone walls

As shown in the table below (Table 2), it was found that most of the stone wall trees had root systems that were not only superficially attached to the wall but also extended to the top or bottom of the wall. In particular, *Ficus* spp. whose strong root system could closely mosaic with the wall, thus forming a strong symbiosis.

**Table 2:** The relationship between the root system of the stone wall tree and the wall.

Location of root growing	count	Frequency (%)	Image
Only superficially attached	5	5.2	
Surface-attached and roots growing in the bottom of wall	42	43.8	
Surface-attached and roots growing in the top of wall	22	22.9	
Surface-attached, roots both growing in the top and bottom of wall	27	28.1	

(photo was taken by Professor Qin Xinsheng).

Stone walls can imitate the traditional nature-accommodating features to permit spontaneous establishment of a diverse plant assemblage. Besides vegetative diversities in terms of species composition, growth form and biomass structure, stone walls can support a mass collection of urban wildlife and provide various ecosystem service. It is highly recommended that modern urban design be created to embrace stone wall landscape as an integral part of naturalistic or ecological design.

### Vision for the establishment of the stone wall tree trail system in the historic of Macao

**Figure 5:**
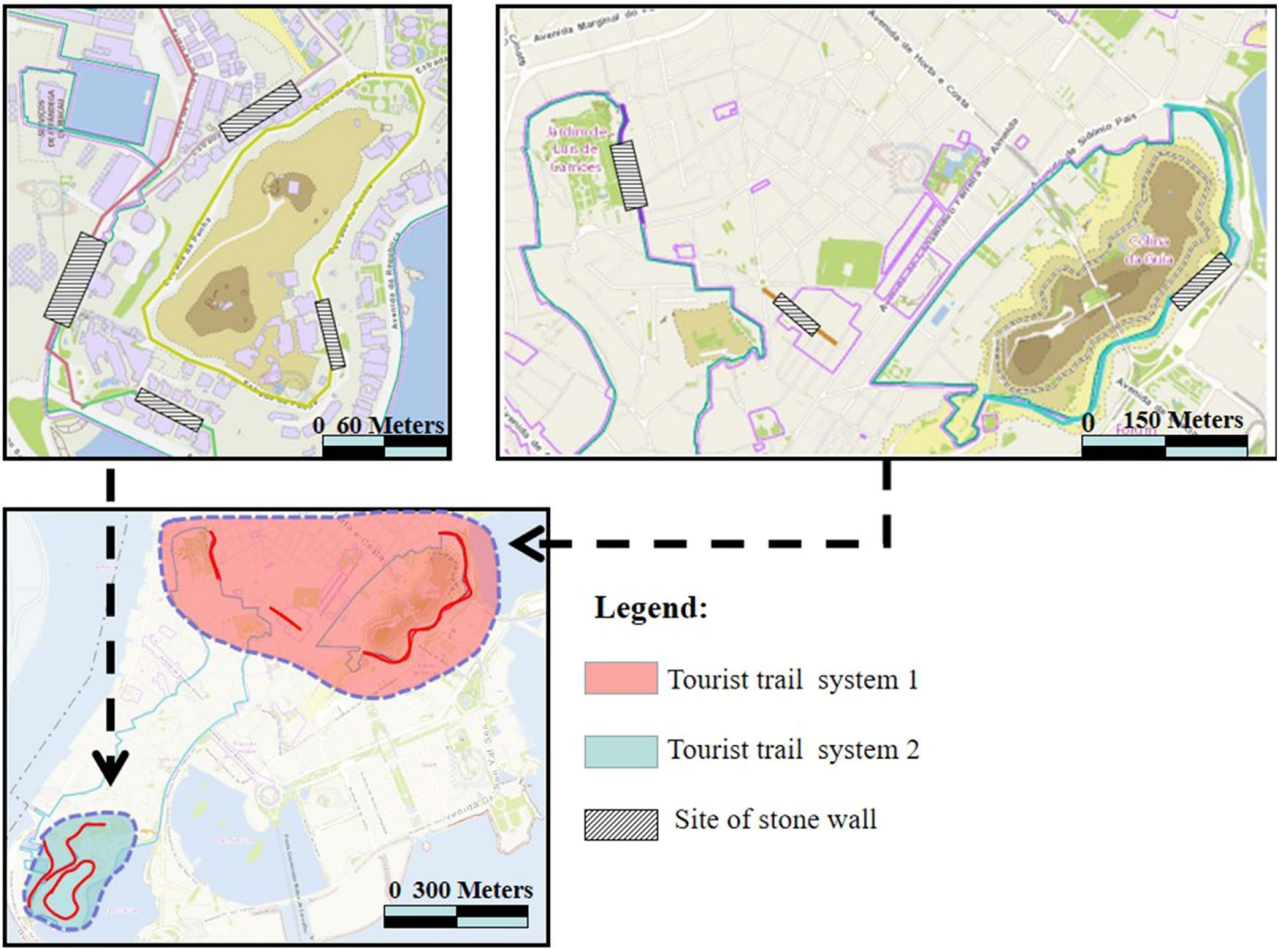
Schematic diagram of the stone wall trees trail system on the Macao Peninsula (URL of the Macao map: https://www.d-maps.com/m/asia/china/macau/macau02.gif and the finished map is created by Meisi Chen through the Photoshop CS6 and Arc GIS 10.2).

The traditional street environment in the Macao Peninsula is a kind of distinctive urban landscape, which can highlight the specificity and value of the urban context. The combination of the stone wall trees and walls, together with the traditional streets, form a spatial urban landscape. Starting from the location of the stone wall tree landscape, the dots and lines are prospective to promote the establishment of a comprehensive stone wall tree landscape trail system (Fig. 5), so that the public can make use of the existing biological resources to have a better understanding of the land on which they live.

Since 2012, the Macao Government has been implementing the "Strolling along Macao Street" project, which aims at studying and exploring the history and culture of the streets of Macao through an in-depth cultural tourism route and promoting it to different levels of society. The establishment of the stone wall tree trail system can rely on this project to raise the public's awareness of the protection and cultural identity of the stone wall tree landscape through a variety of ways. For example, route design competition, photography competition and exhibition, recruitment of "Stonewall Tree Protection Ambassadors" and other forms of participation, so that the public could complete the “role change” in the high degree of such participation—from “onlookers” to “bystanders”.

### Survey results of associated plant species

#### Species composition and occurrence of frequency

The survey showed that there were 101 species of stone wall tree associated plants in Macao, under 88 genera and 51 families. Most associated plants belonged to Euphorbiaceae, Compositae, and Araceae.

**Figure 6:**
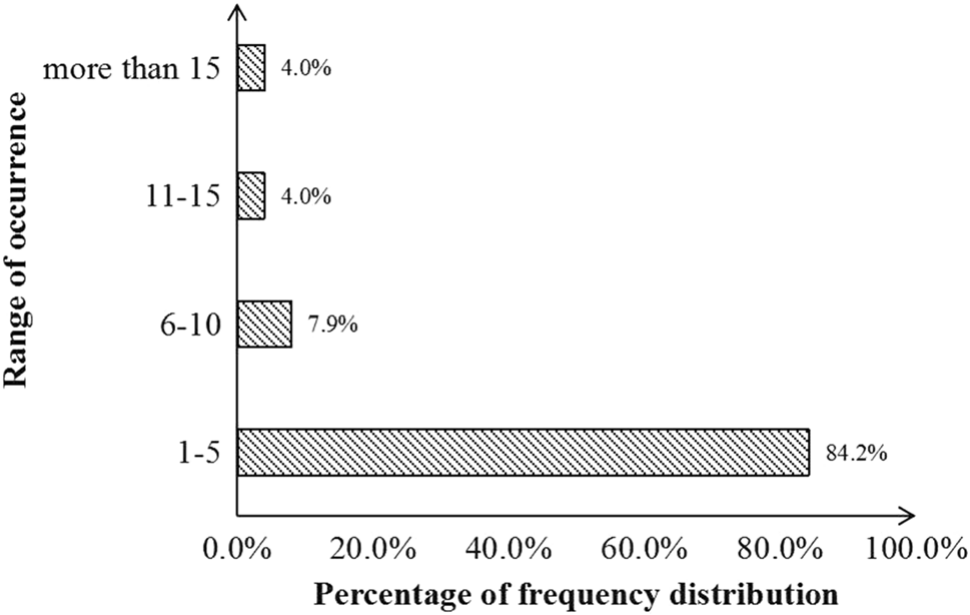
Occurrence frequency in various species of associated plants.

There were 85 species with a frequency of 1–5 times, accounting for 84.2% of total species. A total of 11 species appeared 11–15 times, accounting for 4.0% (Fig. 6). There were a total of 4 species that appeared more than 15 times. They were *Cocculus orbiculatus*, *Pteris cretica*, *Paederia scandens*, and *Pyrrosia adnascens*. Most of the associated species appeared only 1–5 times, indicating that most plants were selective and accidental for the growth conditions of stone wall sites.

#### Life form composition

**Figure 7:**
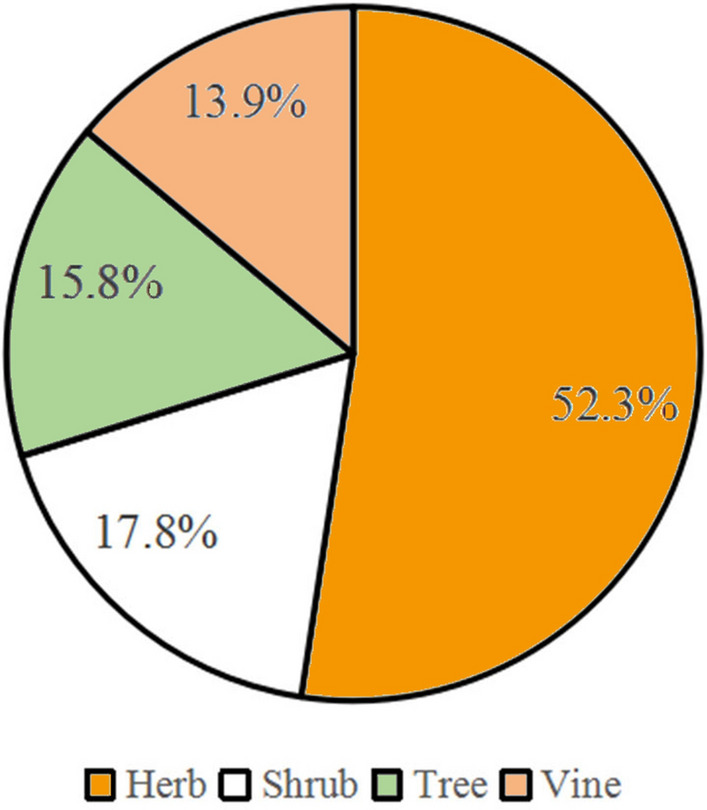
Life form of associated plants with stone wall trees in Macao.

Herbaceous plants with 37 species, accounting the percentage of 52.3% (Fig. 7), were dominant in the associated plant species because the seeds of herbaceous plants are lighter and can be propagated to the wall surface by wind force.

#### Similarity analysis of the associated plants in Macao

In order to compare the similarity of associated plant species in different environment, the surveyed sample sites for this study were divided into three categories: motorized lanes, non-motorized lanes, and park habitats (Table 3). According to Jaccard's similarity principle, S_j_ is extremely dissimilar when it is 0.00–0.25, and the analysis showed that the similarity of companion plant species in all three habitats was extremely dissimilar. Therefore, it indicated that the companion plants in different habitats had obvious diversity and uniqueness.

**Table 3:** Jaccard similarity index for companion plant species composition among three habitats.

Plots	Motorized lanes	Non-motorized lanes	Parks
Motorized lanes			
Non-motorized lanes	0.190		
Parks	0.130	0.042	

## Discussion

### Differences in species composition of stone wall plants between Macao and cities in adjacent climate zones

The growth of stone wall trees was compared between several cities whose climate conditions are similar to Macao. The life form and species composition of the trees on the stone walls in Macao, Hong Kong, Nanjing and Chongqing show regular changes as the climate zone moves southward^21^.

Hong Kong, similar to the climate and environment of the Macao region, also existed extensive stone wall trees. Similar to the survey of the stone wall trees in Macao, *Ficus microcarpa* was also the dominant species of stone wall trees^2^. The reason why it can become widespread group of stone wall trees was closely related to its root vitality. *Ficus microcarpa* could maintain the state of "dry death" in the environment of long-term water shortage^22^. When the rain falls, the aerial roots that seem to be dry and dead for a long time could grow new roots, reflecting a strong vitality. Therefore, compared with other plants, *Ficus microcarpa* may have better adaptability to the barren and dryness on the stone wall.

According to a survey of 289 stone walls in Chongqing^11^, *Ficus virens* was dominant tree species of this city. *Ficus virens* has a long history of urban greening in Chongqing, which enables it to become an absolute dominant tree species in Chongqing. Compared with *Ficus microcarpa*, *Ficus virens* as a kind of deciduous tree is more adaptable to the climate conditions with less precipitation in Chongqing area and is more tolerant to the winter environmental stress in the area.

The dominant tree species in Nanjing was *Broussonetia papyrifera*, which was also the common tree species in Hong Kong, and Chongqing. *Broussonetia papyrifera* with fast-growing characteristics, whose fruits are succulent and large in quantity, are easy to spread seeds through birds^23^.

The dominant stone wall tree species in various regions were closely related to local climate characteristics, natural species resources, characteristics of tree species, and human cultivation history^24^. The combination of different factors had created differences in the dominant stone wall tree species between cities.

### Ecological benefits of stone wall trees in Macao

Most of the stone wall tree groups were *Ficus* species^25^, which were tall in shape and dense in crown. The most frequent occurrence of *Ficus microcarpa* among the *Ficus* was 60.4%. It shows that *Ficus microcarpa* is well adapted to the special growing environment of the stone wall and can absorb the nutrients inside the stone wall through its strong root system, thus ensuring its growth in the difficult environment. *Ficus microcarpa* are excellent habitats for many birds and insects, giving birth to relatively stable animal and plant habitat communities, so as to improve the local microclimate and environment.

### Stone wall trees should be integrally protected together with the wall

The stone walls together with their vegetative companions constitute a unique urban ecology in Macao. The fortunate combination of abiotic and biotic factors, in an inordinately harsh compact city environment, on an apparently inhospitable habitat, has allowed abundant vegetative. Considering the stressful habitat conditions, the resulting of plant diversity is rare surprising, comprising a various range of species and life forms that range from lichen-moss to herbs, shrubs and trees.

The rich diversity and pleasant landscape quality of wall vegetation provide essential environmental and visual amenities. The mural vegetation has thrived spontaneously owing to a century or more of minimal human disturbance. Thus stone wall plants and walls are supposed to be protected as a combination to maximize their ecological benefit and aesthetic value. Measures could be taken to impede the decline in the natural supply of water-nutrient, seeds that could allow plant establishment.

## Conclusions

In the twenty-first century, the development of new technology revolution has brought tremendous changes to the economic structure, people’s living concepts, lifestyles and cultural practices. While enjoying the convenience and comfort brought by high technology, cities also impart people a sense of loneliness and depression. Many start to attach importance to the urban environment which is humane, historical and cultural. This research clarified in detail stone wall trees’ important ecological and cultural value in Macao by analyzing their species characteristics and distribution locations. The combined landscape formed by the stone wall tree and walls is an important element in the study of Macao, as the landscape is closely connected with the adjacent space and the emotions of the residents through time, therefore it is necessary to explore rational and scientific conservation strategies for the stonewall landscape in order to optimize the urban ecosystem.

### Guideline statement

This research strictly complies with the Regulations of the People's Republic of China on the Protection of Wild Plants, and we conducts legal research and utilization of the wild plants involved in the survey.
